# Effects Of 6-Week Telerehabilitation Exercise Programme On Chronic Non-Specific Neck Pain In Women: A Pilot Randomized Controlled Trial

**DOI:** 10.12688/f1000research.160247.1

**Published:** 2025-02-07

**Authors:** Zaina Zahur Ahmed, Mohammad Nayel Aqil Khan, Kalyana Chakravarthy Bairapareddy

**Affiliations:** 1 Department of Physiotherapy, University of Sharjah, Sharjah, Sharjah, United Arab Emirates

**Keywords:** Telerehabilitation, Neck pain, Home exercises

## Abstract

**Background:**

Chronic non-specific neck pain is one of the most prevalent musculoskeletal disorders affecting work and lifestyle. Physiotherapy techniques, such as stretching and strength training, have beneficial effects on neck pain. Telerehabilitation exercise programmes could readily address the growing concern of patient adherence to home-based exercise programmes, while being time- and cost-effective. This study aimed to determine the effectiveness of telerehabilitation exercise intervention in females with chronic non-specific neck pain by measuring the pain score, disability index, cervical range of motion (CROM), cervical muscle endurance, and patient adherence.

**Methods:**

In this pilot randomized controlled trial, 31 females (mean age 22.7 ± 2.1 years) were given a 6-week home-based exercise programme based on their assigned group: telerehabilitation (TR) – online software or conventional (CG) – exercise manual. Baseline measurements were collected using the Visual Analog Scale for Pain (VAS Pain), Neck Disability Index (NDI) questionnaire, CROM using the CROM instrument, and cervical muscle endurance through the Craniocervical Flexion Test (CCFT), and repeated after six weeks, in addition to adherence. SPSS version 26.0 was used for all statistical analyses.

**Results:**

Based on mixed model ANOVA measures (0 week and 6 weeks), within-group comparisons for both groups showed statistical significance in favour of the exercise programme, for all variables (p<0.05). Telerehabilitation group showed significantly more increase in cervical rotation ROM R (0.006) and L (0.03) post-exercise programme, and longer duration of treatment session (0.02) as compared to conventional group. Between-group comparisons showed no significant differences for all other variables.

**Conclusions:**

Based on our findings, both groups showed significant improvement in neck pain, disability, cervical ROM, and cervical muscle endurance; however, no group was found superior to the other in this regard. While both groups showed good adherence to the frequency of sessions, telerehabilitation exhibited better adherence to the duration of the exercise sessions.

## Introduction

Chronic non-specific neck pain is a growing problem affecting almost 50% of adults in the general population. According to Goode et al. (2010), the majority of individuals with this musculoskeletal illness either never respond to treatment or never find a way to manage their pain, which has a detrimental effect on their ability to work and lead a normal life. ^1^ Several studies have identified multiple risk factors for neck pain. These include causes such as excessive physical demands at work, unsuitable workplace conditions, psychosocial factors (including stress and anxiety), sleep deprivation, recurrent chronic low back pain, and female sex. ^2, 3^ In general, females are associated with a higher prevalence of pain when compared to males. This has been linked to various psychosocial influences, including views and approaches concerning health, feeling more disagreeable towards pain, greater exposure to psychological distress, and unintentional prejudice among healthcare providers. ^3, 4^ Moreover, the global burden of neck pain, in terms of years lived with a disability, is far greater in women than in men. ^5^

The benefits of physical therapy on the intensity of pain experienced, degree of disability, and cervical range of motion (CROM) are becoming increasingly evident. Strengthening exercises, endurance exercises, stretching, and postural education are types of training included in these exercise programmes. ^6^ Moreover, active exercise training has been shown to improve the strength of the neck musculature. ^7^ Considering that a large part of the exercise programme consists of self-management, in terms of administering the exercises themselves, education, awareness, and lifestyle changes, ^8^ home-based exercises are recommended, particularly in those who suffer from chronic pain. ^9^ The exercises are taught to the patient, and they are encouraged to follow all the advice provided by the physiotherapist; however, the adherence of patients to these programmes often poses an obstacle to relief from chronic pain. ^10^ Home-based exercise programmes have shown similar improvements in pain, disability, and cervical muscle endurance compared with supervised exercise programmes. ^11, 12^

In recent decades, exercise programmes that are patient-suitable, time-efficient, and cost-effective have become much greater in demand. ^13^ Moreover, in light of the global circumstances raised by the COVID-19 pandemic, telerehabilitation interventions—which are currently getting widely popular—have been chosen in this context. ^14^ The advantages of this method of delivering healthcare have been demonstrated in numerous studies with a particular focus on patient adherence to treatment. ^12^ CareSpace software, in collaboration with the Connect2MyDoctor application, was adopted as a means of telerehabilitation in this study. CareSpace Inc. ^15^ is a US-based multinational digital health company that offers an online platform for patient care management. The facility of this platform to digitize musculoskeletal care via a mobile or webcam powered by visual (AI-powered) learning to measure, record, and recommend personalized care pathways to improve outcomes on the population health scale was utilized based on the requirements of this study for recording exercise sessions.

## Methods

### Study design

This study compared the effectiveness of two different methods of administering the same 6-week home-based exercise programme for chronic nonspecific neck pain. This was a pilot randomized controlled trial. The study participants were randomized by the researcher into two groups: telerehabilitation (TR) or conventional (CG) using the permuted block randomization method. Five permuted blocks were used, each with a block size of six (ABABAB, BABABA, AAABBB, AABBAB, and BBAABA). The co-researcher (KR) administered treatment allocation using sealed and opaque envelopes that were sequentially numbered. Data for the outcome measures (pretest and posttest) were obtained by the lead researcher (ZA) both at the beginning and end of the 6-week period. Due to the nature of the study, blinding of the researchers or participants was not possible.

### Sample

**Figure 1.**
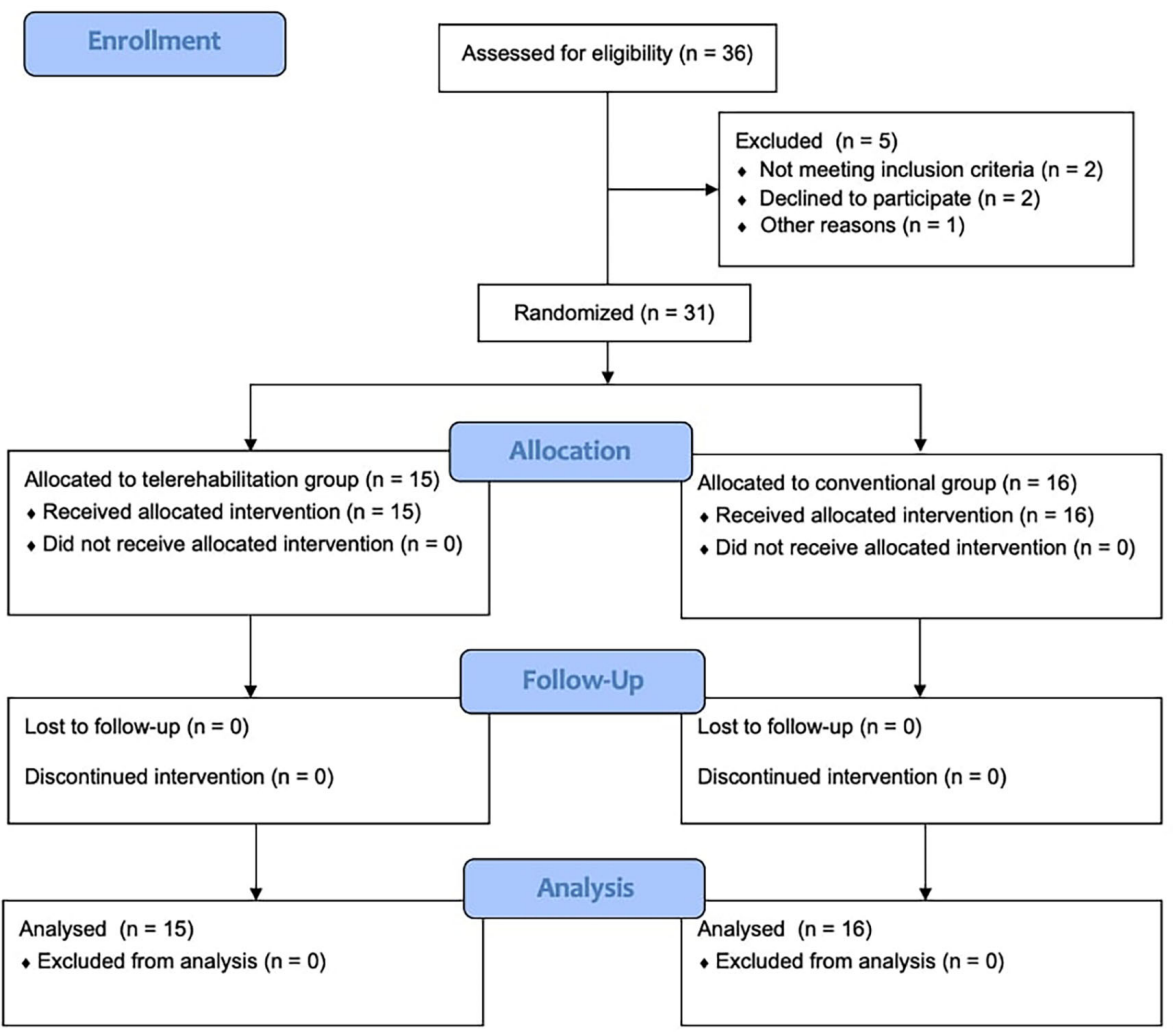
Consolidated standards of reporting trials flow diagram.

Convenience sampling was selected for this study. Considering that this was a pilot study, 30 participants were anticipated (15 for each group). The final study sample included 31 participants (15 in the TR and 16 in the CG), as shown in Figure 1. Recruited participants were included in this study based on the following criteria: (1) residents of the UAE, only females; (2) aged between 18 and 45 years; (3) had normal BMI: 18.5 – 24.9 kg/m ^2 16^; (4) referred to physiotherapy by an orthopedic surgeon for chronic non-specific neck pain; (5) a minimum pain score of 3 cm on the VAS Pain (Visual Analog Scale for Pain); (6) pain onset at least 3 months ago; and (7) provided informed verbal and written consent for voluntary participation. The exclusion criteria were as follows: (1) diagnosis by an orthopedic surgeon with any pathological condition as the cause of chronic neck pain; (2) presently undergoing any physical treatment or taking any medication for said pain; (3) comorbidities such as diabetes and hypertension; (4) conditions such as migraine and cervicogenic headache; (5) vertigo and/or vertebrobasilar insufficiency; (6) physically disabled or recently immobilizing injury; (7) orthopaedic conditions, such as spondylosis, spondylolisthesis, wryneck; (8) current pregnancy; and (9) history of neurological conditions.

### Measures

*Pain*

Pain intensity was self-reported on the VAS Pain, which is a continuous scale presented as a 10 cm line. The scale is ideal for use in this study because it has shown acceptable reliability (0.94) and validity for chronic diseases, ranging from 0.62 to 0.91. ^17^ Pain score was considered the primary outcome.

*Disability*

The NDI (Neck Disability Index) questionnaire was chosen because it is commonly used in research studies to measure neck pain and disability experienced from said pain. The alpha coefficient was found to be 0.08, denoting acceptable reliability and validity of this questionnaire for assessing neck pain and disability. ^18^

*Cervical ROM*

Active ROM of the cervical region was assessed using a CROM instrument. Measurements were obtained using this tool for ROM of cervical flexion, extension, lateral flexion, and rotation to both sides. The CROM instrument consists of compasses for each plane (sagittal, frontal, and horizontal), which are secured to a firm headgear. Hence, this tool was chosen because it shows acceptable reliability and validity for active cervical ROM measurement. ^19^

*Endurance*

For the craniocervical flexion test (CCFT), the participant was tested in the supine position with the head neutral. A pressure biofeedback device was placed under the patient’s neck, with a starting pressure of 20 mmHg. The participant was then asked to perform a chin tuck, which was explained as a gentle head nod. They were instructed to hold the chin tuck for 10 s at five different pressure levels, gradually increasing from 22 to 30 mmHg. ^20^ The highest pressure level maintained by each participant was recorded. Intra- and inter-examiner reliability for this test based on the intraclass coefficient (ICC) was found to be moderate (0.63-0.86). ^21^

*Adherence*

Adherence is a measure of how well a patient can follow professional advice for a home-based exercise programme, including how often (frequency per week) and how long each session is conducted (duration). ^10^ The CG used an exercise diary for self-report adherence. ^22^ For TR, since each session was recorded and stored on the cloud, it was checked and confirmed by the researcher to be reviewed in the biweekly review sessions.

### Exercise programme

Over the course of six weeks, participants were instructed to perform the given exercises on their own at home three times a week, for a total of 18 sessions, 30 minutes each. TR used the CareSpace programme to follow a pre-recorded video. This software enabled them to record and save the entire session on the cloud, so that the therapist could review it. CG were given an exercise manual with clear written instructions, guiding the participant through each exercise, as well as an exercise diary to record the details of each session.

### Procedure

Upon receiving ethical approval from the UOS Research Ethics Committee (REC-23-02-27-01-PG) and registration with ClinicalTrials.gov (US) (NCT06076174), data collection commenced. E-posters with study details were sent out to announce the recruitment of participants, and those who voluntarily offered their participation, met the requirements of the inclusion criteria and gave verbal and written consent were recruited. Prior to the start of the exercise programme, baseline measurements were obtained from all participants in person at the University of Sharjah. These included the pain score on the VAS Pain scale, ^23^ score on the NDI questionnaire, ^18^ CROM measurement using the CROM instrument ^14^ and cervical muscle endurance assessed by the CCFT. ^20^ After collecting the baseline measurements and before starting the programme, the physiotherapist conducted an in-person session at the University of Sharjah, with each participant performing all the exercises. The physiotherapist had received the required training to conduct the demonstration of exercises and monitor and follow up with the participants. The first review session was held by the physiotherapist after two weeks, one-on-one with each participant. For TR, this was done virtually in real time using the Connect2MyDoctor application, and for CG, it was conducted over the phone. Similarly, the second review session took place after four weeks. During both review sessions, the participants performed exercise progressions. Following completion of the 6-week exercise programme, posttest measures were obtained. All assessments were conducted in a private room at the University of Sharjah, and all data were stored on a secure account on a secure computer. Recorded videos of participant sessions of TR were stored in the cloud, and only the lead researcher (female) had access to these recordings. The software provider company or anyone in the university department, other than the lead researcher, did not have access to these data. All patient information and data were kept confidential. No participant name was used for publication or presentation.

### Statistical analysis

The dependent variables were pain score, disability index, CROM, and cervical muscle endurance. These outcome measures were collected from all participants at baseline and upon completion of all exercise sessions, in addition to data regarding adherence to the treatment programme. All statistical analyses were performed using the SPSS (Statistical Package for the Social Sciences) version 26.0. The Shapiro-Wilk test determined that the data had a normal distribution. Frequencies, means, and standard deviations (SD) were calculated using descriptive statistics. An independent samples t-test was conducted to determine baseline differences between the groups. For all within-group and between-group analyses, repeated-measures ANOVA was used at two distinct time points: 0 weeks and 6 weeks. Statistical significance was set at p < 0.05. The effect size statistics for the pretest and posttest mean values for all variables in both groups were calculated using paired sample t-tests. Cohen’s d value benchmark was considered for effect size as small (d = 0.2), medium (d = 0.5), or large (d = 0.8). ^24^ The interpretation of effect size, for between-groups comparisons, was done utilizing the partial eta squared, with thresholds defined as follows: values greater than 0.01 are considered small, those exceeding 0.06 are deemed moderate, and those surpassing 0.14 are classified as large. ^25^

## Results

All 31 participants (females) completed the 6-week intervention (15 in the TR and 16 in the CG). Table 1 presents baseline demographics and participant characteristics. The mean age was 22.7 ± 2.1 years and all participants had a normal BMI (mean 22.3 ± 2.8 kg/m ^2^). Both groups showed no significant differences in the baseline values of demographic data and all outcome measures, as concluded from the independent sample t-tests (p < 0.05). The mean pain score on VAS Pain was 5.2 ± 1.7 cm (5.1 ± 1.9 cm for TR and 5.3 ± 1.5 cm for CG). The mean NDI was 6.3 ± 2.0 (5.7 ± 1.9 for TR and 6.9 ± 1.9 for CG). The Shapiro–Wilk’s test (p<0.05) was performed to confirm the normal distribution of the data.

**Table 1.** Baseline characteristics of the participants (mean ± SD).

Variable	Total sample (N = 31)	TR (N = 15)	CG (N = 16)	P value (p<0.05)
Age (years)	22.7 ± 2.1	22.3 ± 2.5	23 ± 1.7	0.36
Height (cm)	165.4 ± 6.3	164.7 ± 6.3	166.1± 6.4	0.99
Weight (kg)	61.3 ± 10.2	59.5 ± 9.7	63.0 ± 10.6	0.85
BMI (kg/m ^2^)	22.3 ± 2.8	21.9 ± 2.8	22.7 ± 2.7	1.00
VAS Pain (cm)	5.2 ± 1.7	5.1 ± 1.9	5.3 ± 1.5	0.33
NDI	6.3 ± 2.0	5.7 ± 1.9	6.9 ± 1.9	0.91
Cervical Flexion ROM ( ^o^)	42.7 ± 5.9	41.8 ± 5.2	43.6 ± 6.6	0.19
Cervical Extension ROM ( ^o^)	52.1 ± 8.1	49.6 ± 6.4	54.7 ± 9.1	0.22
Cervical R Lateral Flexion ROM ( ^o^)	34.2 ± 5.9	34.9 ± 6.2	33.5 ± 5.6	0.62
Cervical L Lateral Flexion ROM ( ^o^)	38.0 ± 6.2	39.0 ± 4.7	36.9 ± 7.4	0.03
Cervical R Rotation ROM ( ^o^)	51.5 ± 6.9	49.5 ± 5.2	53.6 ± 8.0	0.07
Cervical L Rotation ROM ( ^o^)	53.3 ± 7.0	52.9 ± 6.1	53.7 ± 8.0	0.48
CCFT (mmHg)	25.5 ± 1.8	25.5 ± 1.5	25.5 ± 2.1	0.37

Abbreviations: BMI, Body Mass Index; VAS Pain, Visual Analog Scale for Pain; NDI, Neck Disability Index; ROM, range of motion; R, right; L, left; CCFT, Craniocervical flexion test.

To compare the adherence of the participants to the exercise programme, independent samples t-tests (p < 0.05) were carried out based on the mean changes in the number of sessions completed and the average duration of each session. While the number of sessions completed was not significantly different between the groups, it was found that TR followed through for a significantly longer duration of each session (p = 0.02) than CG ( Table 2).

**Table 2.** Adherence variables between-groups comparison (mean ± SD and 95% confidence interval).

	TR (N = 15)	CG (N = 16)	Between-groups P value (p<0.05)
Number of sessions completed	16.7 ± 2.1	16.5 ± 2.0	0.85
Average duration of each session	23.7 ± 2.4	20.2 ± 4.6	0.02

**Figure 2.**
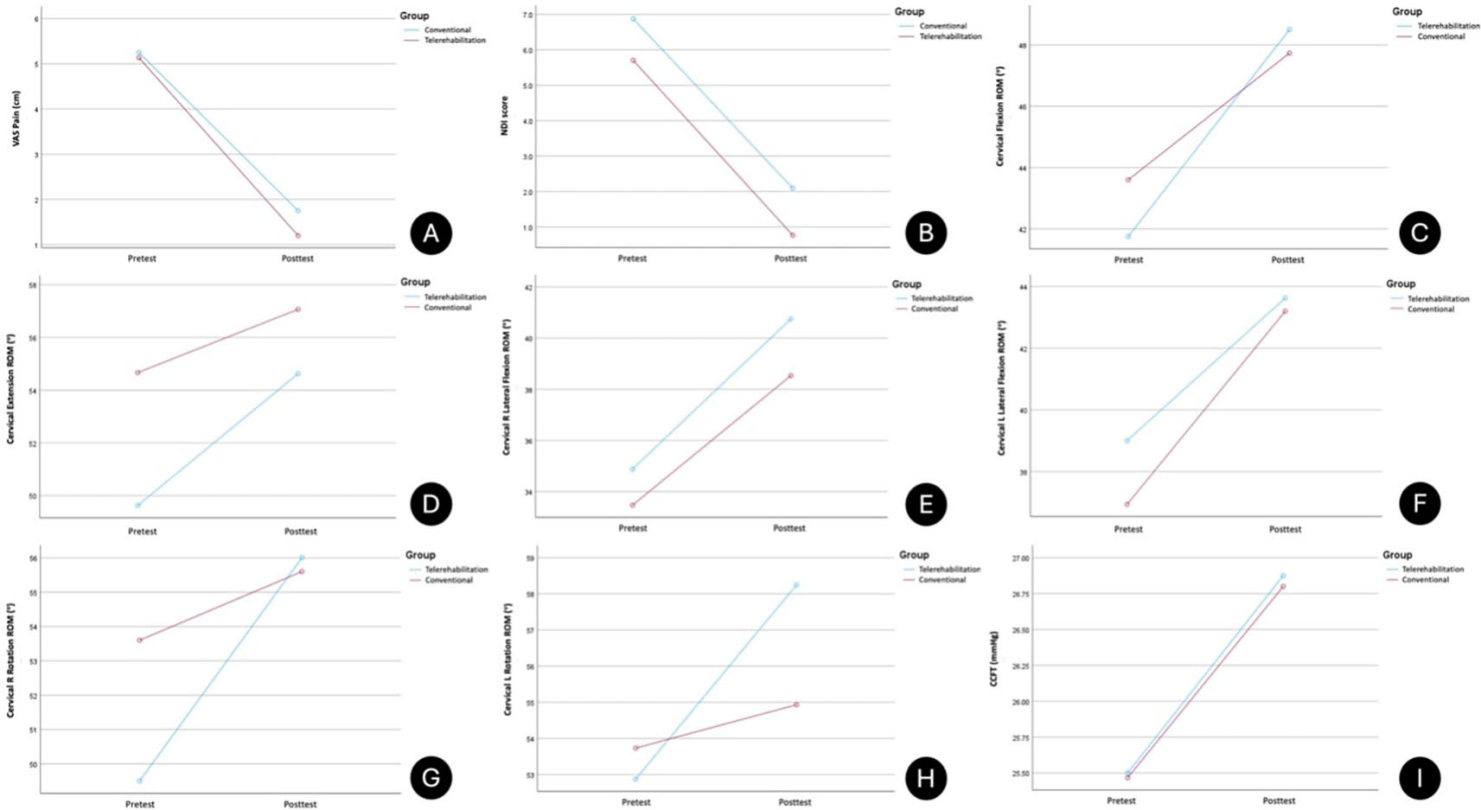
Line graphs depicting profile plots of estimated marginal means. (A) VAS Pain. (B) NDI score. (C) Cervical flexion ROM. (D) Cervical extension ROM. (E) Cervical R lateral flexion ROM. (F) Cervical L lateral flexion ROM. (G) Cervical R rotation ROM. (H) Cervical L rotation ROM. (I) CCFT.

Based on mixed-model ANOVA measures, statistical significance was found in both within-group comparisons, in favor of the exercise programme, for all variables (p < 0.05). TR showed a significantly greater increase in cervical rotation ROM R (0.006) and L (0.03) post-exercise programmes than CG. Between-group comparisons of all other variables showed no significant differences. A large, significant effect on pain score, disability index, cervical flexion, right lateral flexion, and right rotation ROM was found in both groups post-treatment, whereas medium effects on cervical extension, left lateral flexion and left rotation ROM, and cervical muscle endurance were observed. When looking at between-groups values, large effect on pain score (0.14) and disability index (0.17) was found in favor of telerehabilitation ( Table 3). Line graphs depicting the profile plots of the estimated marginal means for all the variables are shown in Figure 2.

**Table 3.** Within-group and between-groups changes for outcome measures (mean ± SD and 95% confidence interval) post-treatment.

		Pretest	Posttest	Effect size (Cohen’s d, 95% CI) Within-group	Effect size (ηp2 ^a^) Between-groups	P value (p<0.05)	
						Within-group	Between-groups
**VAS Pain (cm)**	*TR*	5.1 ± 1.9	1.2 ± 1.0	2.20 (1.23, 3.14)	0.14	<.001	0.47
	*CG*	5.3 ± 1.5	1.8 ± 2.0	2.33 (1.36, 3.28)		<.001	
**NDI**	*TR*	5.7 ± 1.9	0.8 ± 0.7	3.02 (1.80, 4.22)	0.17	<.001	0.84
	*CG*	6.9 ± 1.9	2.1 ± 2.0	2.02 (1.14, 2.87)		<.001	
**Cervical Flexion ROM (** ^**o**^ **)**	*TR*	41.8 ± 5.2	48.5 ± 5.9	-1.10 (-1.71, -0.46)	0.03	<.001	0.14
	*CG*	43.6 ± 6.6	47.7 ± 5.9	-1.44 (-2.16, -0.69)		0.003	
**Cervical Extension ROM (** ^**o**^ **)**	*TR*	49.6 ± 6.4	54.6 ± 11.1	-0.49 (-1.00,0.34)	0.05	0.01	0.35
	*CG*	54.7 ± 9.1	57.1 ± 55.8	-0.79 (-1.36, -0.19)		0.23	
**Cervical R Lateral Flexion ROM (** ^**o**^ **)**	*TR*	34.9 ± 6.2	40.8 ± 7.1	-1.92 (-2.75, -1.07)	0.03	<.001	0.54
	*CG*	33.5 ± 5.6	38.5 ± 5.2	-1.20 (-1.87, -0.52)		<.001	
**Cervical L Lateral Flexion ROM (** ^**o**^ **)**	*TR*	39.0 ± 4.7	43.6 ± 9.4	-0.76 (-1.31, -0.19)	0.01	0.03	0.58
	*CG*	36.9 ± 7.4	43.2 ± 7.9	-0.63 (-1.18, -0.07)		0.006	
**Cervical R Rotation ROM (** ^**o**^ **)**	*TR*	49.5 ± 5.2	56.0 ± 6.8	-1.24 (-1.89, -0.57)	0.02	<.001	0.006
	*CG*	53.6 ± 8.0	55.6 ± 7.4	-0.76 (-1.33, -0.17)		0.07	
**Cervical L Rotation ROM (** ^**o**^ **)**	*TR*	52.9 ± 6.1	58.25 ± 7.3	-0.81 (-1.37, -0.23)	0.01	<.001	0.03
	*CG*	53.7 ± 8.0	54.9 ± 9.0	-0.40 (-0.92, -0.13)		0.38	
**CCFT (mmHg)**	*TR*	25.5 ± 1.5	26.8 ± 1.9	-0.73 (-1.27, -0.16)	<.001	0.01	0.95
	*CG*	25.5 ± 2.1	26.8 ± 1.8	-0.64 (-1.18, -0.07)		0.02	

Abbreviations: TR, Telerehabilitation Group; CG, Conventional; BMI, Body Mass Index; VAS Pain, Visual Analog Scale for Pain; NDI, Neck Disability Index; ROM, Range of Motion; R, right; L, left; CCFT, Craniocervical flexion test.

^a^ Partial eta squared.

## Discussion

This study aimed to investigate the effects of home-based exercise programmes for chronic non-specific neck pain, based on the mode of delivery. Comparisons were made based on neck pain, disability, cervical ROM, cervical muscle endurance, and adherence to the exercise programme. It is known that physiotherapy interventions consisting of strengthening and stretching exercises result in the reduction of neck pain and disability, as well as an enhancement in cervical ROM and cervical muscle endurance. ^26– 28^

Based on our findings, both groups showed significant improvements in neck pain upon successful completion of the prescribed home-based exercise programme. In addition to improvement in self-reported pain, disability experienced as assessed by the NDI also reduced, similar to the findings of previous studies conducted on individuals with chronic non-specific neck pain, by utilizing telerehabilitation. ^11, 29^ Moreover, when comparisons were drawn between the results of both groups, the telerehabilitation group proved to be non-inferior to the conventional group in positively influencing neck pain and disability. ^30, 31^

Cervical ROM was assessed in terms of cervical flexion, extension, lateral flexion, and rotation on both sides. ^32^ Post-exercise measurements for both groups exhibited an increase in all values of cervical ROM, similar to findings from other studies. ^3, 33^ Both groups presented a significant increase in cervical muscle endurance based on the findings of CCFT; however, no group was found superior to the other in this regard. This finding is in line with that of a study conducted by Ozer et al. (2021). Notably when it comes to improving posture and relieving chronic neck pain, exercises that target the activation of the deep flexor muscle of the neck (such as chin tucks and chin tucks with overpressure) have demonstrated superior outcomes compared to general strengthening exercises. ^34^ This is explained by findings that reveal that cervical muscles that have undergone strength training in addition to endurance training and stretching demonstrate increases in motor unit recruitment, firing rate per unit, and capillarization, all of which result in an increase in the strength and endurance of the cervical musculature. ^7, 35^ Our findings suggest that telerehabilitation provides meaningful clinical improvement in patient symptoms, unlike previous studies. ^13^ Healthcare providers and patients alike can be encouraged to consider this mode of treatment delivery when access, time, and costs pose difficulties. ^8, 31^

Regarding adherence to the exercise programme, both methods of treatment delivery were feasible since, overall, both groups showed good compliance with the frequency of sessions (3 times a week for 6 weeks). When looking at adherence to session duration, our study found that TR had longer sessions on average than CG. This may be owed to the video recording and monitoring facilities available from the telerehabilitation software that enable the therapist to review the entire session. ^10, 36, 37^ However, both groups exhibited shorter exercise durations than the recommended duration of 30 minutes, ^38^ possibly due to time constraints, busy lifestyles, or experiencing improvement in symptoms even with a shorter session duration.

The effect of confounders cannot be ignored when comparing all variables, particularly pain, as it was the main outcome measure in this study. The diet of an individual is a variable confounding factor that can affect the neck pain experienced by them, which was not anticipated. ^39, 40^ Another such factor is sleeping position, as it is known to greatly affect the intensity of pain, especially waking pain and perceived severity of disability. ^41, 42^ Additionally, anxiety and depression have been identified as psychosocial factors that alter the pain perception of those affected by it. ^43, 44^ Physical activity and chronic pain sensitivity have also been associated in recent studies, particularly in women. ^45, 46^ Moreover, some participants may have used a cervical collar without informing the researcher, which may have influenced the amount of pain felt.

Raising awareness of healthy and active lifestyles is key to a person’s general well-being and is crucial in managing any type of chronic pain. Particularly in cases where people find healthcare inaccessible owing to distance, time, or high costs, telerehabilitation-based care can prove to be quite effective and long-lasting. ^12, 47^ All participants were given general advice after completing the 6-week exercise programme. These included ergonomic training, education about sleeping and sitting posture, and advice to continue with recommended exercises from the home programme, along with general fitness topics. ^9, 47^ Considering that a large proportion of participants in this age group spent long hours using a computer, sitting at a desk posture was explained to them, such as the use of a spinal support chair, distance between eye and monitor, adjusting the desk height so that the forearms could rest on the surface, adjusting the chair height so that the feet could rest on the floor, etc. ^48, 49^ Moreover, the importance of comfortable sleeping posture in either supine or side-lying, with appropriate neck support, was elaborated, in addition to the use of a good pillow with the right amount of thickness (10-12 cm) and firmness. ^42, 50^

For those suffering from chronic non-specific neck pain, especially females, this mode of exercise delivery brings about the success of exercise sessions in terms of symptom improvement as well as increased cervical muscle ROM and endurance. Based on these results, especially considering adherence, treatment is favorable for TR. Telerehabilitation, due to its benefits of increased patient adherence, cost-effectiveness, and providing convenience, gives medical professionals with a robust means to providing healthcare. Although it proves to be a useful tool for home-based exercises, it is crucial to remember that telerehabilitation is recommended as an extension to in-person care and not a replacement for hands-on clinical sessions, such as manual therapy, provided by a physiotherapist. Blinding of neither participants nor researchers was done, which presents a limitation. Because all participants in this study were females, the results cannot be generalized to the entire population. Moreover, all participants who were randomized into the telerehabilitation group were expected to have software-compatible devices, such as laptops or mobile phones, as well as a stable internet connection. The duration of treatment may be another potential limitation. Considering that this is a pilot study, a larger sample size, longer duration of treatment, and extended follow-up periods for randomized controlled trials are recommended for the future. Studies can build on the methods used in this study, and in addition to its findings, by including both gender comparisons, exploring the effects of varying treatment durations is recommended. Furthermore, the influence of factors such as diet, physical activity, sleeping position, and stress should also be studied.

### Ethics and consent

Upon receiving ethical approval from the UOS Research Ethics Committee (REC-23-02-27-01-PG) and registration with ClinicalTrials.gov (US) (NCT06076174), data collection commenced. E-posters with study details were sent out to announce the recruitment of participants, and those who voluntarily offered their participation, met the requirements of the inclusion criteria and gave verbal and written consent were recruited.

## Data Availability

Dataset and extended date are available in Harvard Dataverse repository, under the title “Effects of 6-Week Exercise Programme On Chronic Non-Specific Neck Pain In Women”, DOI: https://doi.org/10.7910/DVN/ZMC9DU, under the CC0 1.0 license. ^51^

## References

[1] Goode AP Freburger J Carey T : Prevalence, practice patterns, and evidence for chronic neck pain. *Arthritis Care Res (Hoboken).* 2010;62(11):1594–1601. 10.1002/acr.20270 20521306 PMC2974793

[2] Kääriä S Laaksonen M Rahkonen O : Risk factors of chronic neck pain: A prospective study among middle-aged employees. *Eur. J. Pain (United Kingdom).* 2012;16(6):911–920. 10.1002/j.1532-2149.2011.00065.x 22337254

[3] Kazeminasab S Nejadghaderi SA Amiri P : Neck pain: global epidemiology, trends and risk factors. *BMC Musculoskelet. Disord.* 2022;23(1):13–26. 10.1186/s12891-021-04957-4 34980079 PMC8725362

[4] Palacios-Ceña D Albaladejo-Vicente R Hernández-Barrera V : Female Gender Is Associated with a Higher Prevalence of Chronic Neck Pain, Chronic Low Back Pain, and Migraine: Results of the Spanish National Health Survey, 2017. *Pain Med (United States).* 2021;22(2):382–395. 10.1093/pm/pnaa368 33164071

[5] Shin DW Shin JI Koyanagi A : Global, regional, and national neck pain burden in the general population, 1990–2019: An analysis of the global burden of disease study 2019. *Front. Neurol.* 2022;13:955367. 10.3389/fneur.2022.955367 36119688 PMC9477009

[6] Bertozzi L Gardenghi I Turoni F : Effect of therapeutic exercise on pain and disability in the management of chronic nonspecific neck pain: Systematic review and meta-analysis of randomized trials. *Phys. Ther.* 2013;93(8):1026–1036. 10.2522/ptj.20120412 23559524

[7] Borisut S Vongsirinavarat M Vachalathiti R : Effects of strength and endurance training of superficial and deep neck muscles on muscle activities and pain levels of females with chronic neck pain. *J. Phys. Ther. Sci.* 2013;25(9):1157–1162. 10.1589/jpts.25.1157 24259936 PMC3818764

[8] Fandim JV Costa LOP Yamato TP : Telerehabilitation for neck pain. *Cochrane Database Syst. Rev.* 2021;2021(3). 10.1002/14651858.CD014428 PMC12341027 40792483

[9] Khosrokiani Z Letafatkar A Hadadnezhad M : The comparison between the effects of pain education interventions with online and face-to-face exercise and the control group received biomedical education + standardized physical therapy in patients with chronic nonspecific neck pain during COVID-19: pr. *Trials.* 2022;23(1):1–11. 10.1186/s13063-022-06932-3 36539843 PMC9763812

[10] Medina-Mirapeix F Escolar-Reina P Gascán-Cnovas JJ : Predictive factors of adherence to frequency and duration components in home exercise programs for neck and low back pain: An observational study. *BMC Musculoskelet. Disord.* 2009;10(1):1–9. 19995464 10.1186/1471-2474-10-155 PMC2796992

[11] Gialanella B Ettori T Faustini S : Home-Based Telemedicine in Patients with Chronic Neck Pain. *Am. J. Phys. Med. Rehabil.* 2017;96(5):327–332. 10.1097/PHM.0000000000000610 27584139

[12] Ozer AY Kapsigay B Şenocak E : Effectiveness of different exercise programs in individuals with non-specific neck pain: telerehabilitation, given with synchronous exercises versus homebased exercise. *Med. Sport. J. Rom. Sport. Med. Soc.* 2021;17(2):3327–3335. Reference Source

[13] Cottrell MA O’Leary SP Raymer M : Does telerehabilitation result in inferior clinical outcomes compared with in-person care for the management of chronic musculoskeletal spinal conditions in the tertiary hospital setting? A non-randomised pilot clinical trial. *J. Telemed. Telecare.* 2021;27(7):444–452. 10.1177/1357633X19887265 31771410

[14] Wahezi S Duarte RA Yerra S : Telemedicine during covid-19 and beyond: A practical guide and best practices multidisciplinary approach for the orthopedic and neurologic pain physical examination. *Pain Physician.* 2020;4S;23(4 Special Issue):S205–S237. 10.36076/ppj.2020/23/S205 32942812

[15] CareSpace, Inc: Reference Source

[16] Zierle-Ghosh A Jan A : Physiology, Body Mass Index. *StatPearls.* 2024. 30571077

[17] Lemeunier N Silva-Oolup S da Olesen K : Reliability and validity of self-reported questionnaires to measure pain and disability in adults with neck pain and its associated disorders: part 3—a systematic review from the CADRE Collaboration. *Eur. Spine J.* 2019;28:1156–1179. 10.1007/s00586-019-05949-8 30879185

[18] Vernon H Mior S : The Neck Disability Index: A study of reliability and validity. *J. Manip. Physiol. Ther.* 1991;14:409–415. Vernon, Howard: 1900 Bayview Avenue, Toronto, ON, Canada, M4G 3E6: Mosby Publishing Company. 1834753

[19] Yee Won YK : The Reliability and Validity on Measuring Tool of Cervical Range of Motion: A Review. *Sport. Med. Inj. Care J.* 2019;1(1):1–4. 10.24966/SMIC-8829/100001

[20] De Araujo FX Ferreira GE Schell MS : Measurement properties of the craniocervical flexion test: A systematic review. *Phys. Ther.* 2020;100(7):1094–1117. 10.1093/ptj/pzaa072 32313944

[21] Jørgensen R Ris I Falla D : Reliability, construct and discriminative validity of clinical testing in subjects with and without chronic neck pain. *BMC Musculoskelet. Disord.* 2014;15(1):1–15. 25477032 10.1186/1471-2474-15-408 PMC4325947

[22] Nicolson PJA Hinman RS Wrigley TV : Self-reported home exercise adherence: A validity and reliability study using concealed accelerometers. *J. Orthop. Sports Phys. Ther.* 2018;48(12):943–950. 10.2519/jospt.2018.8275 30053792

[23] Hawker GA Mian S Kendzerska T : Measures of adult pain: Visual Analog Scale for Pain (VAS Pain), Numeric Rating Scale for Pain (NRS Pain), McGill Pain Questionnaire (MPQ), Short-Form McGill Pain Questionnaire (SF-MPQ), Chronic Pain Grade Scale (CPGS), Short Form-36 Bodily Pain Scale (SF). *Arthritis Care Res.* 2011;63(SUPPL. 11):240–252. 10.1002/acr.20543 22588748

[24] Cohen J : *Statistical Power Analysis for the Behavioral Sciences.* 2nd Ed. Hillsdale, NJ: Lawrence Erlbaum Associates;1988.

[25] Richardson JTE : Eta squared and partial eta squared as measures of effect size in educational research. *Educ. Res. Rev.* 2011;6(2):135–147. 10.1016/j.edurev.2010.12.001

[26] Akhter S Khan M Ali SS : Role of manual therapy with exercise regime versus exercise regime alone in the management of non-specific chronic neck pain. *Pak. J. Pharm. Sci.* 2014;27(6):2125–2128. 25410083

[27] Häkkinen A Kautiainen H Hannonen P : Strength training and stretching versus stretching only in the treatment of patients with chronic neck pain: A randomized one-year follow-up study. *Clin. Rehabil.* 2008;22(7):592–600. 10.1177/0269215507087486 18586810

[28] Salo PK Häkkinen AH Kautiainen H : Effect of neck strength training on health-related quality of life in females with chronic neck pain: A randomized controlled 1-year follow-up study. *Health Qual. Life Outcomes.* 2010;8:1–7. 20465854 10.1186/1477-7525-8-48 PMC2877013

[29] De Zoete RMJ Armfield NR McAuley JH : Comparative effectiveness of physical exercise interventions for chronic non-specific neck pain: A systematic review with network meta-analysis of 40 randomised controlled trials. *Br. J. Sports Med.* 2021;55(13):730–742. 10.1136/bjsports-2020-102664 33139256

[30] Onan D Ulger O Martelletti P : Effects of spinal stabilization exercises delivered using telerehabilitation on outcomes in patients with chronic neck pain: a randomized controlled trial. *Expert. Rev. Neurother.* 2023;23(3):269–280. 10.1080/14737175.2023.2192870 36927237

[31] Zou H Lu Z Zhao P : Efficacy of telerehabilitation in patients with nonspecific neck pain: A meta-analysis. *J. Telemed. Telecare.* 2024. 10.1177/1357633X241235982 38425292

[32] MacDermid JC : Use of Outcome Measures in Managing Neck Pain: An International Multidisciplinary Survey. *Open Orthop. J.* 2013;7(1):506–520. 10.2174/1874325001307010506 24115972 PMC3793628

[33] Hidalgo B Hall T Bossert J : The efficacy of manual therapy and exercise for treating non-specific neck pain: A systematic review. *J. Back Musculoskelet. Rehabil.* 2017;30(6):1149–1169. 10.3233/BMR-169615 28826164 PMC5814665

[34] Young Kim J il Kwag K : Clinical effects of deep cervical flexor muscle activation in patients with chronic neck pain. *J. Phys. Ther. Sci.* 2016;28:269–273. 10.1589/jpts.28.269 26957772 PMC4756018

[35] Kashfi P Karimi N Peolsson A : The effects of deep neck muscle-specific training versus general exercises on deep neck muscle thickness, pain and disability in patients with chronic non-specific neck pain: protocol for a randomized clinical trial (RCT). *BMC Musculoskelet. Disord.* 2019;20(1):1–8. 31727085 10.1186/s12891-019-2880-x PMC6857347

[36] Bennell KL Marshall CJ Dobson F : Does a Web-Based Exercise Programming System Improve Home Exercise Adherence for People with Musculoskeletal Conditions?: A Randomized Controlled Trial. *Am. J. Phys. Med. Rehabil.* 2019;98(10):850–858. 10.1097/PHM.0000000000001204 31021823

[37] Özden F Özkeskin M Tümtürk İ : The effect of exercise and education combination via telerehabilitation in patients with chronic neck pain: A randomized controlled trial. *Int. J. Med. Inform.* 2023;180:105281. 10.1016/j.ijmedinf.2023.105281 37924590

[38] O’Riordan C Clifford A Van De Ven P : Chronic neck pain and exercise interventions: Frequency, intensity, time, and type principle. *Arch. Phys. Med. Rehabil.* 2014;95(4):770–783. 10.1016/j.apmr.2013.11.015 24333741

[39] Elma Ö Yilmaz ST Deliens T : Chronic Musculoskeletal Pain and Nutrition: Where Are We and Where Are We Heading? *Am. Acad. Phys. Med. Rehabil.* 2020;12(12):1268–1278. 10.1002/pmrj.12346 32086871

[40] Torlak MS Gonulalan G Tufekci O : The effect of therapeutic exercise and vegan diet on pain and quality of life in young female patients with chronic non-specific neck pain. *Bull. Fac. Phys. Ther.* 2022;27(1). 10.1186/s43161-021-00061-9

[41] Cary D Jacques A Briffa K : Examining relationships between sleep posture, waking spinal symptoms and quality of sleep: A cross sectional study. *PLoS One.* 2021;16(11):e0260582–e0260513. 10.1371/journal.pone.0260582 34847195 PMC8631621

[42] Chun-Yiu JP Man-Ha ST Chak-Lun AF : The effects of pillow designs on neck pain, waking symptoms, neck disability, sleep quality and spinal alignment in adults: A systematic review and meta-analysis. *Clin. Biomech.* 2021;85(April):105353. 10.1016/j.clinbiomech.2021.105353 33895703

[43] Angst F Benz T Lehmann S : Extended overview of the longitudinal pain-depression association: A comparison of six cohorts treated for specific chronic pain conditions. *J. Affect. Disord.* 2020;273(1):508–516. 10.1016/j.jad.2020.05.044 32560947

[44] Wong KP Tse MMY Qin J : Effectiveness of Virtual Reality-Based Interventions for Managing Chronic Pain on Pain Reduction, Anxiety, Depression and Mood: A Systematic Review. *Healthcare.* 2022;10(10). 10.3390/healthcare10102047 36292493 PMC9602273

[45] Lee MK Oh J : The relationship between sleep quality, neck pain, shoulder pain and disability, physical activity, and health perception among middle-aged women: a cross-sectional study. *BMC Womens Health.* 2022;22(1):110–186. 10.1186/s12905-022-01773-3 35597981 PMC9124008

[46] Skogberg O Karlsson L Börsbo B : Pain Tolerance in Chronic Pain Patients Seems To Be More Associated With Physical Activity Than With Depression and Anxiety. *J. Rehabil. Med.* 2022;54(7):jrm00286. 10.2340/jrm.v54.241 35274145 PMC9131201

[47] Özlü A Ünver G Tuna Hİ : Effects of Interactive Telerehabilitation Practices in Office Workers with Chronic Nonspecific Neck Pain: Randomized Controlled Study. *Telemed. e-Health.* 2024;30(2):438–447. 10.1089/tmj.2023.0018 37498517

[48] Alshehre YM Pakkir Mohamed SH Nambi G : Effectiveness of Physical Exercise on Pain, Disability, Job Stress, and Quality of Life in Office Workers with Chronic Non-Specific Neck Pain: A Randomized Controlled Trial. *Healthcare.* 2023;11(16). 10.3390/healthcare11162286 37628484 PMC10454597

[49] Johnston V Chen X Welch A : A cluster-randomized trial of workplace ergonomics and neck-specific exercise versus ergonomics and health promotion for office workers to manage neck pain – a secondary outcome analysis. *BMC Musculoskelet. Disord.* 2021;22(1):1–12. 33435941 10.1186/s12891-021-03945-y PMC7805092

[50] Yamada S Hoshi T Toda M : Changes in neck pain and somatic symptoms before and after the adjustment of the pillow height. *J. Phys. Ther. Sci.* 2023;35(2):106–113. 10.1589/jpts.35.106 36744195 PMC9889209

[51] Ahmed Z : Effects of 6-Week Exercise Programme On Chronic Non-Specific Neck Pain In Women. *Harvard Dataverse.* 2024. 10.7910/DVN/ZMC9DU

