# Supplementary material for: Effects Of 6-Week Telerehabilitation Exercise Programme On Chronic Non-Specific Neck Pain In Women: A Pilot Randomized Controlled Trial
Source: F1000Res. 2025 Apr 22;14:174. Originally published 2025 Feb 7. [Version 2] doi: 10.12688/f1000research.160247.2 (PMC12442751; doi:10.12688/f1000research.160247.2)
Supplement: Peer review history

# Reviewer report: Reviewer response for version 1

**DOI:** 10.5256/f1000research.176111.r377585

**Published:** 2025-05-02

**Authors:** Jie Hao

**Affiliations:** 1 University of Nebraska Medical Center, Omaha, USA

The authors have conducted a well-structured pilot randomized controlled trial comparing a telerehabilitation intervention with a conventional home-based exercise program. The study design is clearly described, and the manuscript includes appropriate outcome measures such as pain, disability, cervical ROM, muscle endurance, and adherence. While the manuscript is overall well-written, several issues should be addressed to enhance the clarity of the study.

 The manuscript refers to this as a "pilot study," but it lacks a clear rationale or reference for the selected sample size of 30 participants. Even in pilot trials, providing justification for the sample size is essential to contextualize findings.

 The rationale for studying only female participants should be better justified.

 Please explicitly state the research hypotheses and expected outcomes.

Is the work clearly and accurately presented and does it cite the current literature?

Yes

If applicable, is the statistical analysis and its interpretation appropriate?

Yes

Are all the source data underlying the results available to ensure full reproducibility?

Yes

Is the study design appropriate and is the work technically sound?

Yes

Are the conclusions drawn adequately supported by the results?

Yes

Are sufficient details of methods and analysis provided to allow replication by others?

Yes

Reviewer Expertise:

physical therapy, digital health, rehabilitation

I confirm that I have read this submission and believe that I have an appropriate level of expertise to confirm that it is of an acceptable scientific standard, however I have significant reservations, as outlined above.

# Reviewer report: Reviewer response for version 1

**DOI:** 10.5256/f1000research.176111.r365949

**Published:** 2025-04-11

**Authors:** Animesh Hazari

**Affiliations:** 1 Gulf Medical University, Ajman, United Arab Emirates

Strengths of the Manuscript: The use of randomized controlled trial (RCT) design strengthens the validity of findings. A detailed methodology section ensures reproducibility.

 Telerehabilitation is an emerging field, and the study addresses a timely issue, especially given the post-pandemic shift towards digital healthcare.

 Areas for Improvement and Suggestions:

 1. Abstract: The conclusion states that no group was found superior to the other, & yet partial eta squared values indicate a large effect in favor of telerehabilitation for pain and disability.

 2. Introduction: The manuscript mentions that females have a higher prevalence of neck pain, but additional justification is needed. Expand on sex-specific physiological differences that might influence treatment response.

 3. Methods: The study mentions a permuted block randomization method but does not specify how the block sizes were determined.

 The manuscript states that this is a pilot study but does not provide a rationale for selecting 30 participants.

 4. Results: The between-group p-values for VAS Pain and NDI are non-significant (0.47, 0.84), yet partial eta-squared values suggest a meaningful effect. Consider discussing whether the study was underpowered to detect between- group differences.

 5. Discussion: The discussion mostly reiterates results but should provide more actionable insights for clinicians.

 While the discussion mentions diet, sleep, and psychological factors as confounders, the study does not account for these variables. Suggested to acknowledge this as a limitation and propose future research to explore these effects.

 6. Conclusion: The conclusion states that telerehabilitation is a viable alternative but does not emphasize its benefits in adherence.

Is the work clearly and accurately presented and does it cite the current literature?

Yes

If applicable, is the statistical analysis and its interpretation appropriate?

Partly

Are all the source data underlying the results available to ensure full reproducibility?

Partly

Is the study design appropriate and is the work technically sound?

Yes

Are the conclusions drawn adequately supported by the results?

Yes

Are sufficient details of methods and analysis provided to allow replication by others?

Yes

Reviewer Expertise:

Biomechanics

I confirm that I have read this submission and believe that I have an appropriate level of expertise to confirm that it is of an acceptable scientific standard.

# Reviewer report: Reviewer response for version 1

**DOI:** 10.5256/f1000research.176111.r367541

**Published:** 2025-02-25

**Authors:** İsmet Tümtürk

**Affiliations:** 1 Antalya Bilim University, Antalya, Turkey

First of all, I congratulate the authors for this valuable work. However, I would like to make some suggestions.

 Introduction

 1- Please state the purpose and hypotheses of the study.

 2- Elaborate the introduction a little more, e.g. with a description of the advantages of tele rehabilitation and the importance of the study method among these advantages. Indicate the contribution of the study to the literature.

 Methods

 1- Check the manuscript with regard to English. Write sentences more clearly and simply. For example, “This study compared the effectiveness of two different methods of administering the same 6-week home-based exercise program for chronic nonspecific neck pain. This was a pilot randomized controlled trial.” can be corrected to "The present pilot randomized controlled trial investigated the effectiveness of 6-week tele rehabilitation program in women with chronic nonspecific neck pain".

 2- How did you determine the sample size? If you used a method similar to G*Power, please specify this in detail.

 3- Give the explanations for abbreviations such as VAS, ROM, etc. at the first mention in the manuscript.

 4- Detail the measurement procedures. For example, indicate what kind of questions the NDI contains and what its high scores mean, how CROM is measured and cite the procedure used from the literature. 

 5- Please detail the procedure section. What was the sequence of the measurements? Were rest periods given between measurements? What were the exercises given? Which outcome measures were evaluated in the interim assessments and how? Why did the participants in the TR group take videos of themselves during the exercise and in which assessment were these recorded videos used? The procedure section should be detailed enough to adequately inform the readers.

 6- In the statistical analysis part, did the distributions follow a normal distribution (parametric or non-parametric)? It seems that the number of participants in the groups is small, but the statistical methods used are the statistical methods used in parametric distributions. Please clarify this.

 7- ANOVA was used in the statistical analysis, but there are only two groups in the study, and you have specified two measurement points as measurement points (pretest and posttest). ANOVA is mostly used for three or more groups or measurement points. Explain why you preferred ANOVA in this study.

 Results

 1-“All 31 participants (females) completed the 6-week intervention (15 in the TR and 16 in the CG).” in the sentence ‘31’ is redundant.

 2- The baseline characteristics of the participants did not include information such as smoking and symptom duration. If these assessments are available, please add them to Table 1. If not available, discuss in the limitations section.

 Discussion

 1- In the discussion section, discuss the results, including effect sizes.

 2- Write the conclusion as the last paragraph.

 3- Briefly discuss also the results that are not significantly different.

Is the work clearly and accurately presented and does it cite the current literature?

Yes

If applicable, is the statistical analysis and its interpretation appropriate?

Yes

Are all the source data underlying the results available to ensure full reproducibility?

Yes

Is the study design appropriate and is the work technically sound?

Yes

Are the conclusions drawn adequately supported by the results?

Yes

Are sufficient details of methods and analysis provided to allow replication by others?

Partly

Reviewer Expertise:

Orthopedic rehabilitation

I confirm that I have read this submission and believe that I have an appropriate level of expertise to confirm that it is of an acceptable scientific standard, however I have significant reservations, as outlined above.

# Response

**Published:** 2025-03-07

**Authors:** Zaina Ahmed

**Affiliations:** Physiotherapy, University of Sharjah, Sharjah, Sharjah, United Arab Emirates

Introduction

 1- Please state the purpose and hypotheses of the study.

- The following text will be added to the introduction: “The purpose of this study is to determine the effectiveness of telerehabilitation exercise intervention is in females with chronic non-specific neck pain, via measuring the pain score, disability index, CROM, cervical muscle endurance and their adherence to the exercise programme. It is hypothesised that there would be notable improvements in pain score, disability index, CROM, cervical muscle endurance in both groups suffering from chronic non-specific neck pain, but telerehabilitation exercise programme would result in greater improvements in all outcome measures, and particularly better patient adherence, as compared to conventional exercise programme.”

2- Elaborate the introduction a little more, e.g. with a description of the advantages of tele rehabilitation and the importance of the study method among these advantages. Indicate the contribution of the study to the literature.

- The following text will be added to the introduction: “A potential solution to patients not adhering to prescribed exercise regimens could be telerehabilitation. Due to its advantages in terms of patient adherence, cost-effectiveness, and convenience, healthcare professionals are encouraged to implement this type of intervention, which might be extended to in-patient care. For individuals suffering from chronic non-specific neck pain, telerehabilitation can guarantee the effectiveness of following exercise sessions and, consequently, of the treatment programme itself.”

Methods

 1- Check the manuscript with regard to English. Write sentences more clearly and simply. For example, “This study compared the effectiveness of two different methods of administering the same 6-week home-based exercise program for chronic nonspecific neck pain. This was a pilot randomized controlled trial.” can be corrected to "The present pilot randomized controlled trial investigated the effectiveness of 6-week tele rehabilitation program in women with chronic nonspecific neck pain".

- Thank you for your comment, agreed.

2- How did you determine the sample size? If you used a method similar to G*Power, please specify this in detail.

- The following text will be added to the methods - sample: “Based on the effect size of 0.95 utilizing the VAS mean and standard deviation values, alpha error of 5%, study power of 80%, and allocation ratio of 1, a total sample size of 30 participants was deemed suitable (Ozer et al., 2021).”

3- Give the explanations for abbreviations such as VAS, ROM, etc. at the first mention in the manuscript.

- These abbreviations have been expanded wherever they first appear in the manuscript.

4- Detail the measurement procedures. For example, indicate what kind of questions the NDI contains and what its high scores mean, how CROM is measured and cite the procedure used from the literature. 

- Sure, will add what the scores mean.

5- Please detail the procedure section. What was the sequence of the measurements? Were rest periods given between measurements? What were the exercises given? Which outcome measures were evaluated in the interim assessments and how? Why did the participants in the TR group take videos of themselves during the exercise and in which assessment were these recorded videos used? The procedure section should be detailed enough to adequately inform the readers.

- The sequence of measures is as they appear in text. Regarding, the exercise manual, with descriptions for all exercises, are provided as an additional document.

6- In the statistical analysis part, did the distributions follow a normal distribution (parametric or non-parametric)? It seems that the number of participants in the groups is small, but the statistical methods used are the statistical methods used in parametric distributions. Please clarify this.

- It is mentioned in the manuscript that “The Shapiro-Wilk test determined that the data had a normal distribution”.

7- ANOVA was used in the statistical analysis, but there are only two groups in the study, and you have specified two measurement points as measurement points (pretest and posttest). ANOVA is mostly used for three or more groups or measurement points. Explain why you preferred ANOVA in this study.

- The mixed model ANOVA was chosen as it is more flexible and allows us to analyze the effects of continuous within- and between-subjects variables.

Results

 1-“All 31 participants (females) completed the 6-week intervention (15 in the TR and 16 in the CG).” in the sentence ‘31’ is redundant.

- Sure, will change that.

2- The baseline characteristics of the participants did not include information such as smoking and symptom duration. If these assessments are available, please add them to Table 1. If not available, discuss in the limitations section.

- Symptom duration has been mentioned in the inclusion criteria.

Discussion

 1- In the discussion section, discuss the results, including effect sizes.

- Thank you for your comment, will add that.

2- Write the conclusion as the last paragraph.

- Okay, will separate the text into a separate paragraph.

3- Briefly discuss also the results that are not significantly different.

- This has been discussed in the second and third paragraphs of the discussion.
